# Twist1 Influences the Expression of Leading Members of the IL-17 Signaling Pathway in HER2-Positive Breast Cancer Cells

**DOI:** 10.3390/ijms222212144

**Published:** 2021-11-10

**Authors:** Bruno R. B. Pires, Renata Binato, Gerson M. Ferreira, Stephany Corrêa, Bárbara Du Rocher, Daniel Bulzico, Susanne Crocamo, Everton Cruz dos Santos, Luize G. Lima, Eliana Abdelhay

**Affiliations:** 1 Laboratório de Célula-Tronco, Instituto Nacional de Câncer José Alencar Gomes da Silva, Rio de Janeiro 20230-130, RJ, Brazil; rebinato2@hotmail.com (R.B.); gmferreirag@gmail.com (G.M.F.); stephy_correa@yahoo.com.br (S.C.); evertoncruzsantos@gmail.com (E.C.d.S.); 2 Instituto Nacional de Ciência e Tecnologia para o Controle do Câncer, Rio de Janeiro 20230-130, RJ, Brazil; 3 Laboratório de Pesquisa sobre o Timo, Instituto Oswaldo Cruz, Fundação Oswaldo Cruz, Rio de Janeiro 21040-360, RJ, Brazil; barbaradurocher@gmail.com; 4 Unidade de Endocrinologia Oncológica, Instituto Nacional de Câncer José Alencar Gomes da Silva, Rio de Janeiro 20230-130, RJ, Brazil; danielbulzico@gmail.com; 5 Núcleo de Pesquisa Clínica, Hospital de Câncer III, Instituto Nacional de Câncer José Alencar Gomes da Silva, Rio de Janeiro 20560-121, RJ, Brazil; crocamo@inca.gov.br; 6 Tumour Microenvironment Laboratory, QIMR Berghofer Medical Research Institute, Herston, QLD 4006, Australia; Luize.GoncalvesLima@qimrberghofer.edu.au

**Keywords:** breast cancer, HER2, Twist1, IL17, gene expression

## Abstract

Breast cancer (BC) is a heterogeneous disease composed of multiple subtypes with different molecular characteristics and clinical outcomes. The metastatic process in BC depends on the transcription factors (TFs) related to epithelial-mesenchymal transition (EMT), including the master regulator Twist1. However, its role beyond EMT in BC subtypes remains unclear. Our study aimed to investigate the role of Twist1, beyond EMT, in the molecular subtypes of BC. In patients, we observed the overexpression of TWIST1 in the HER2+ group. The silencing of TWIST1 in HER2+ BC cells resulted in the upregulation of 138 genes and the downregulation of 174 genes compared to control cells in a microarray assay. In silico analysis revealed correlations between Twist1 and important biological processes such as the Th17-mediated immune response, suggesting that Twist1 could be relevant for IL-17 signaling in HER2+ BC. IL-17 signaling was then examined, and it was shown that TWIST1 knockdown caused the downregulation of leading members of IL-17 signaling pathway. Taken together, our findings suggest that Twist1 plays a role on IL-17 signaling in HER2+ BC.

## 1. Introduction

Breast cancer (BC) is the leading cause of cancer-related death among women worldwide. In the U.S., approximately 282,000 new cases of female BC and more than 40,000 BC-related deaths are expected in 2021 [1]. Nevertheless, BC is a heterogeneous disease composed of different molecular profiles and diverse clinical outcomes [2]. The main BC subtypes include: (1) Luminal A, which comprises tumors that overexpress estrogen and/or progesterone hormone receptors (ER and PR, respectively); (2) HER2+, consisting of tumors that overexpress the human epidermal receptor 2 (HER2/neu); (3) Luminal B, which includes tumors that express both hormonal and HER2/neu receptors; and (4) triple-negative breast cancer (TNBC) [3]. Over the past two decades, the molecular investigation of BC has increased our understanding of various biological aspects; however, relevant issues regarding the role of the crucial transcription factors for the progression of each intrinsic subtype remain to be addressed.

Late diagnosis increases the risk of invasion and metastasis, which are responsible for 90% of deaths due to carcinomas, including BC [4]. Metastasis is a complex and multistep process in which cancer cells acquire phenotypic features that trigger their ability to migrate and colonize distant organs [5]. Strong evidence suggests that epithelial-to-mesenchymal transition (EMT) plays a pivotal role in tumor metastasis by inducing epithelial cells to adopt a mesenchymal phenotype, thereby leading to metastatic behavior. These changes include the loss of epithelial markers such as E-cadherin, and the acquisition of mesenchymal markers, such as fibronectin [6,7]. This phenotypic transition is regulated by transcription factors (EMT-TFs) [8], with Twist1 being described as “the master regulator of metastasis” in BC [9]. However, the specific role of Twist1 in the different BC subtypes has yet to be examined.

Twist1 is a member of the basic helix-loop-helix (bHLH) transcription factor family that can regulate activating or repressing the expression of target genes through its binding to specific DNA sequences known as E-box (CANNTG) motifs. Twist1 regulates developmental processes in many organs and has been implicated in various carcinomas including BC [10,11,12,13,14,15,16]. However, the role of Twist1 beyond EMT has been poorly investigated in BC. We recently reported that nuclear factor kappa B (NF-κB) acts as a transcriptional regulator of Twist1, Slug, and Sip1 [17]. Based on these results, we sought to examine the expression of these EMT-TFs in BC samples, and their association with clinicopathologic information. We observed the overexpression of Twist1 in the HER2+ group, which led us to investigate the role of Twist1 in this subtype. To this end, we silenced Twist1 in HER2+ BC cells and conducted a microarray analysis. We report here that Twist1 influences the IL-17 signaling in HER2+ BC. Altogether, our results suggest a positive crosstalk between these two signaling pathways, thereby improving the understanding of the relationship between HER2+ BC and immune/inflammatory signaling.

## 2. Results

### 2.1. TWIST1 Is Overexpressed in the HER2+ BC Group

**Figure 1:**
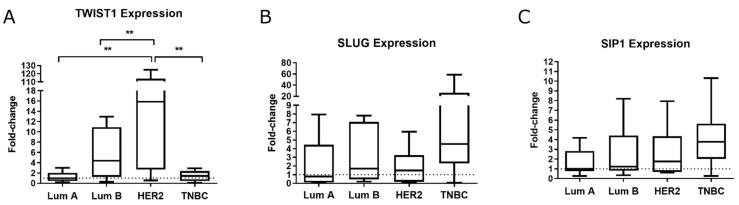
Box-plot graph of *TWIST1* (**A**)*, SLUG/SNAIL2* (**B**) and *SIP1/ZEB2* (**C**) expression (fold-change) in the BC subtypes Luminal A (Lum A), Luminal B (Lum B), HER2+ and Triple-negative (TNBC) from the Brazilian National Cancer Institute cohort. Data are shown as the median (±SD). ** *p* < 0.01.

We assessed the mRNA levels of *TWIST1*, *SLUG*, and *SIP1* in human breast tumor samples from the Brazilian National Cancer Institute cohort. We observed that HER2+ tumors expressed significantly more *TWIST1* than that of other subtypes (Figure 1A). We also found elevated levels of *SLUG* and *SIP1* in TNBC, although the differences were not statistically significant (Figure 1B,C, respectively).

**Figure 2:**
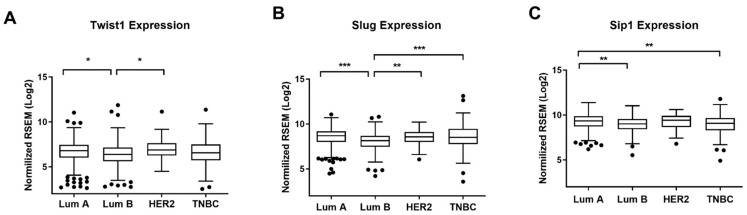
Box-plot graph of *TWIST1* (**A**), *SLUG/SNAI2* (**B**), and *SIP1/ZEB2* expression (**C**) [RNA-Seq by Expectation-Maximization (RSEM), Log2(Val+1)] in Luminal A, Luminal B, HER2+ and triple-negative (TNBC) subtypes. Data are shown as the median (±SD), dots represent outliers. Expression values were obtained from TCGA through Linkedomics tools. * *p* < 0.05; ** *p* < 0.01; *** *p* < 0.001.

We also conducted gene expression analysis at the mRNA level of the EMT-TFs using The Cancer Genome Atlas (TCGA) database for breast invasive carcinoma cohort (TCGA-BRCA, *n* = 1093, RNAseq data normalized counts) through the LinkedOmics portal (http://www.linkedomics.org/login.php; accessed on May 2021). In accordance with the previous results, *TWIST1* expression was higher in the HER2+ group (Figure 2A). However, the difference compared to the other subtypes was lower than what was observed for the Brazilian NCI cohort. The levels of *SLUG* and *SIP* were lower in Luminal B group, and with similar levels for the other subtypes (Figure 2B,C). Together, our findings demonstrate augmented levels of *TWIST1* in HER2+ BC subtype in the Brazilian NCI cohort, and that there are differences between the Brazilian and the TCGA cohorts, perhaps due to ethnic genetic variability.

### 2.2. TWIST1 Knockdown Altered the Gene Expression Profile of Signaling Pathways Relevant to BC

**Figure 3:**
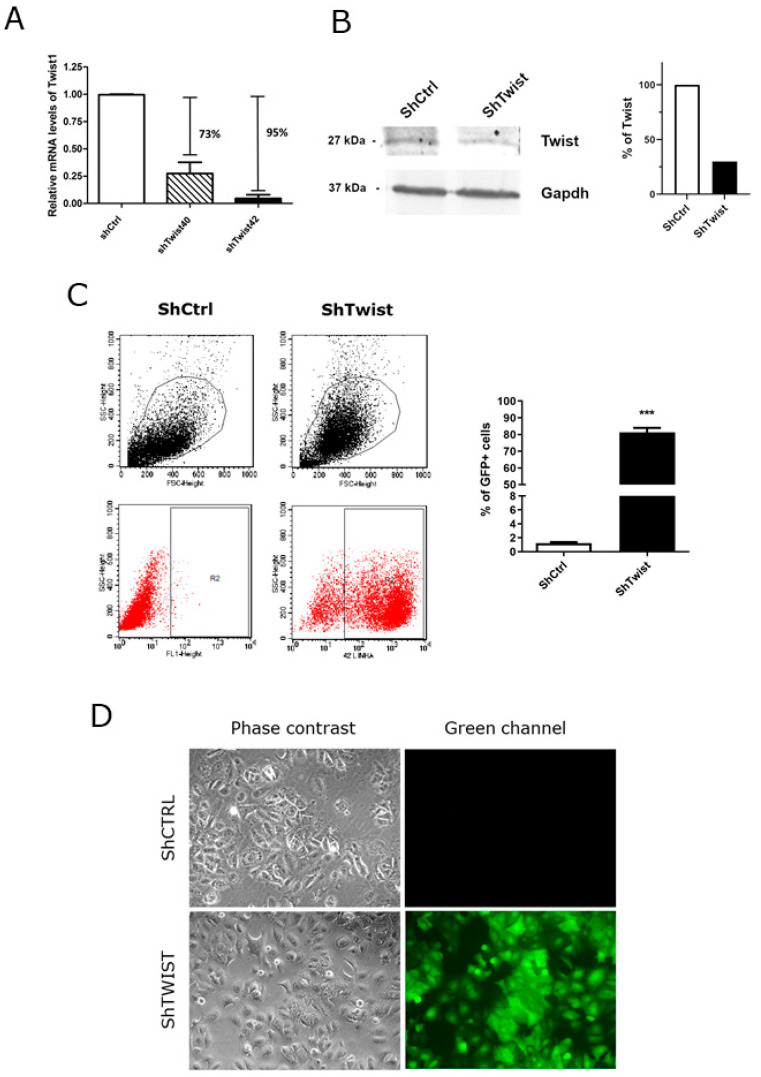
*TWIST1* knockdown in HCC-1954 HER2+ BC cells. *TWIST1* (**A**) mRNA and (**B**) protein levels in response to gene silencing. GAPDH was used as a loading control. Densitometric analysis was done using ImageJ software version 1.53k (**C**) Flow cytometry comparing ShCTRL and ShTWIST cells. The bar graph represents the percentage of GFP positivity. (**D**) Phase-contrast and fluorescence microscopy of ShCTRL and ShTWIST. Magnification is 200×. ShCTRL = negative silencing control; ShTWIST40 and ShTWIST42 are two silenced clones obtained experimentally. Data are shown as the mean (±SD). *** *p* < 0.001.

Since *TWIST1* was shown to be upregulated in HER2+ BC patients, we asked what the biological role of this TF in this subtype was. To answer that, we used the shRNA strategy to knockdown *TWIST1* in a HER2+ BC cell line that expresses significant endogenous levels of this TF. After selection with puromycin, we obtained two transfection clones that showed a significant downregulation of TWIST1 expression at the mRNA level (Figure 3A). Clone #42, which showed a reduction of 95% in *TWIST1* levels (Figure 3A), was used for all subsequent experiments. Using this model (called ShTWIST), *TWIST1* silencing was also confirmed at the protein level (Figure 3B). Because ShTWIST constructs contain the coding sequence for GFP, the expression of GFP was verified by flow cytometry and fluorescence microscopy (Figure 3C,D, respectively). Together, our results confirmed that *TWIST1* was efficiently silenced in our model.

To understand the consequences of *TWIST1* knockdown in HER2+ BC cells, mRNA samples were submitted to microarray analysis. Using a two-fold change as the cutoff for the identification of differentially expressed genes, we found 138 upregulated genes, and 174 downregulated genes in ShTWIST-cells compared to control cells. We then conducted in silico analysis using the MetaCore^TM^ software Version 2019 (http://portal.genego.com/; accessed on May 2019) that associated several genes modulated by *TWIST1* silencing with important biological processes and signaling pathways in BC. Most of these genes participate in more than one relevant biological process (Table 1). Confirming the well-documented role of Twist1 in cancer, the functional enrichment analysis described EMT as the most altered biological process. Importantly, extracellular matrix (ECM) remodeling and blood coagulation were well represented among the differentially expressed genes. Interestingly, our in silico results also identified the T helper 17 (Th17) cell-mediated immune response as one of the best represented biological processes, suggesting that Twist1 is relevant for IL-17 signaling in HER+2 BC. Other relevant in silico data are presented as Supplementary Material (Supplementary Figures S1–S5).

**Figure 4:**
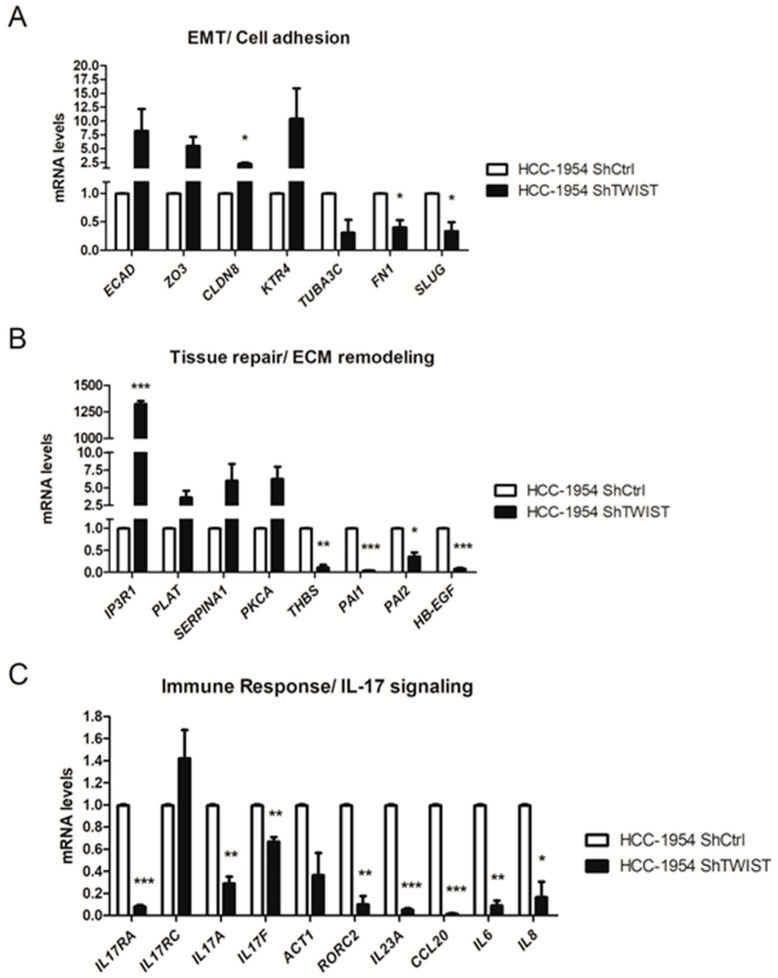
RT-qPCR of the genes altered in the best represented biological processes: (**A**) epithelial-mesenchymal transition (EMT)/Cell adhesion, (**B**) extracellular matrix (ECM) remodeling/Blood coagulation, and (**C**) immune response/IL-17 signaling. ShCTRL = negative silencing control; ShTWIST = TWIST1-knockdown. Data are shown as the mean (±SD). * *p* < 0.05; ** *p* < 0.01; *** *p* < 0.001.

**Figure 5:**
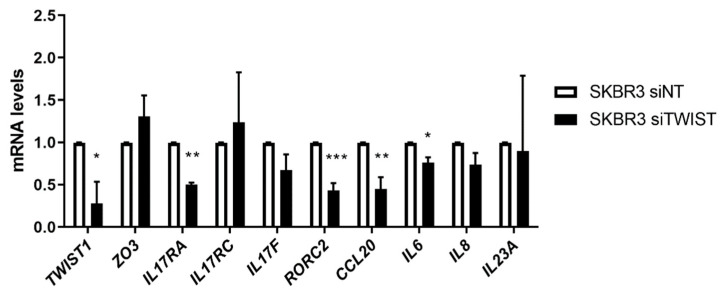
RT-qPCR of the genes altered in consequence of TWIST1 knockdown in SKBR3 cell line. siNT = nontargeting control; siTWIST = TWIST1-silencing using siRNA. Data are shown as the mean (±SD). * *p* < 0.05; ** *p* < 0.01; *** *p* < 0.001.

Using RT-qPCR, we confirmed the expression of selected genes based on the microarray and in silico findings. We also included some relevant members of the IL-17 signaling pathway in this investigation. As shown in Figure 4, the RT-qPCR results corroborated the gene expression profile found in the microarray analysis. We confirmed the upregulation of transcripts encoding epithelial markers, and the downregulation of transcripts corresponding to mesenchymal markers (Figure 4A). Changes in the expression profile of genes related to ECM remodeling/blood coagulation, such as tissue plasminogen activator (*PLAT*), α1-antitrypsin (*SERPINA1*), and plasminogen activator inhibitors 1 and 2 (*PAI1* and *PAI2*), were also confirmed (Figure 4B). Although there is a lack of information regarding the association between Twist1 and immune signaling in BC, the IL-17 signaling pathway was identified as an important target for *TWIST1* knockdown. We found relevant members of this signaling pathway to be downregulated, including the corresponding genes for the major IL-17 subunit receptors *IL17RA* and *IL17RC*, and their immediately downstream molecule, Act1, the cytokines IL-17A, IL-17F, IL-23A, CCL20, IL-6 and IL-8, and the retinoic acid-related orphan receptor (ROR) variant γt (RORC2), which is the most essential TF associated with the Th17 phenotype (Figure 4C). In addition, we confirmed the effects of TWIST1-knockdown in another HER2+ BC cell line, SKBR3 (Figure 5), using a siRNA-approach. Because of the increasing relevance of IL-17 signaling in cancer biology, we focused our investigation on the role of Twist1 in this signaling pathway.

### 2.3. Twist1 Influences Leading Members of the IL-17 Signaling Pathway

**Figure 6:**
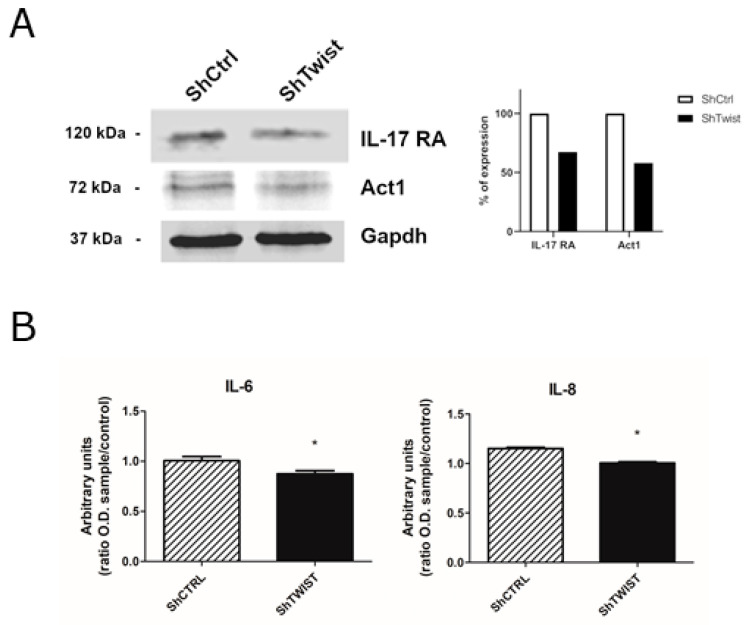
Evaluation of IL-17 signaling in TWIST1-knockdown BC cells compared to control cells. (**A**) Protein levels of Act1 and IL-17RA in ShCTRL and ShTWIST cells. GAPDH was used to ensure that the same sample amounts were loaded for immunoblotting. Densitometric analysis was done using ImageJ software. (**B**) Levels of secreted IL-6 and IL-8 proteins in the supernatant of ShCTRL and ShTWIST cells. Data are shown as the mean (±SD). * *p* < 0.05.

We confirmed the reduction of IL-17RA and Act1 at the protein level (Figure 6A), as well as the decrease of secreted IL-6 and IL-8 proteins in the supernatant of ShTWIST cells (Figure 6B), which are downstream targets of IL-17 signaling. Later, we questioned whether RORγt expression, which is the main TF responsible for the regulation of Th17 differentiation [18], would be associated with the HER2+ subtype, as our previous results indicated.

**Figure 7:**
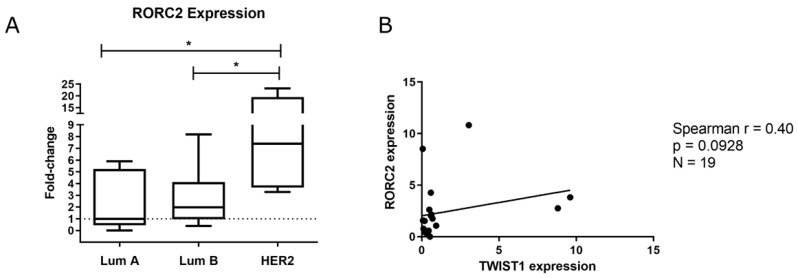
Expression of the variant RORC2 (RORγt) in BC samples from the Brazilian National Cancer Institute cohort. (**A**) Box-plot graph of RORC2 (RORγt) expression in Luminal, Luminal-B and HER2+ subtypes. (**B**) Spearman correlation analysis of the mRNA levels of TWIST1 × RORC2 in HER2+ BC samples. Data are shown as the median (±SD). * *p* < 0.05.

**Figure 8:**
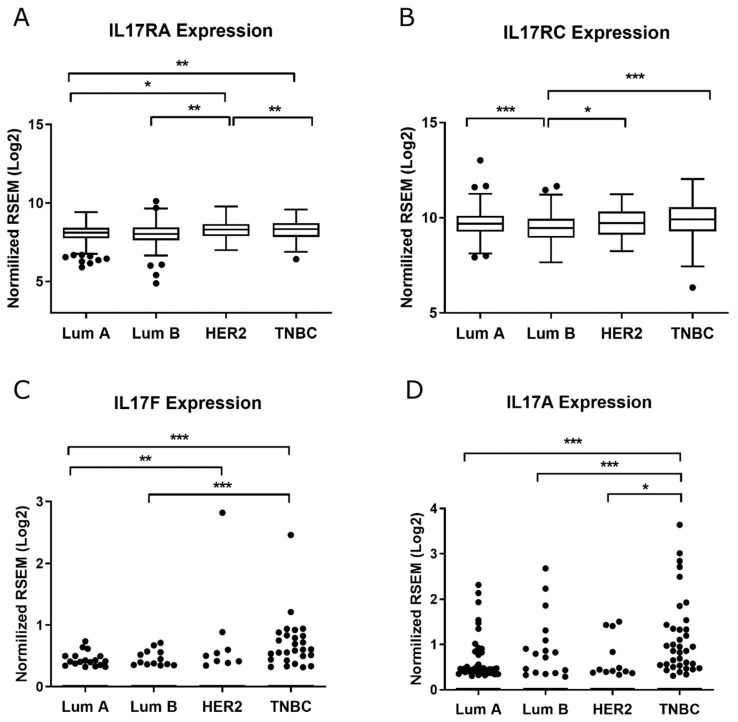
Box-plot graph of IL17RA (**A**), IL17RC (**B**), IL17F (**C**), and IL17A (**D**) expression [RNA-Seq by Expectation-Maximization (RSEM), Log2(Val+1)] in Luminal A, Luminal B, HER2+ and Triple-negative (TNBC) subtypes. Expression values were obtained from TCGA through Linkedomics tools. Data are shown as the median (± SD), dots represent outliers. * *p* < 0.05; ** *p* < 0.01; *** *p* < 0.001.

**Figure 9:**
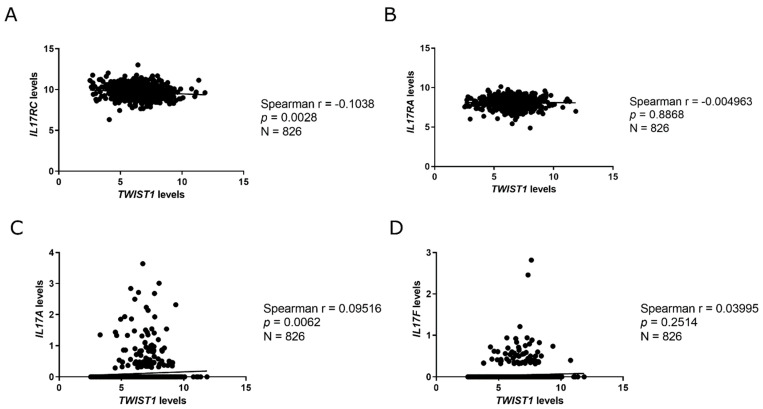
Spearman correlation analysis of the mRNA expression of TWIST1, IL17RC, IL17RA, IL17A, and IL17F in BC samples. Expression values were obtained from TCGA through Linkedomics tools. (**A**) TWIST1 × IL17RC, (**B**) TWIST1 × IL17RA, (**C**) TWIST1×IL17A, (**D**) TWIST1 × IL17F.

We evaluated the mRNA levels of RORγt/RORC2 in the Brazilian National Cancer Institute (Rio de Janeiro, RJ, Brazil) cohort, since the expression of this variant could not be found in the Linkedomics/TCGA database, and we observed that it is overexpressed in HER2+ BC samples (Figure 7A). We also found a positive correlation between the mRNA levels of RORγt/RORC2 and TWIST1 in the HER2+ BC samples (Figure 7B). Further, we analyzed the expression of IL-17 signaling members (IL17RA, IL17RC, IL17A and IL17F) in TCGA database through the LinkedOmics portal and observed that HER2+ tumors expressed significantly more IL17RA, although IL17A was expressed more in TNBC samples (Figure 8). We also performed a correlation analysis between the mRNA levels of TWIST1 and IL-17 signaling members. Corroborating our previous findings, we found a positive correlation between TWIST1 and IL17A expression, and a negative correlation between TWIST1 and IL17RC (Figure 9). Hence, our findings support the positive regulation of IL-17 signaling in HER2+ BC.

## 3. Discussion

Twist1 is an evolutionarily conserved TF initially described as an essential regulator of gastrulation in *Drosophila* sp. [19], but which also plays a critical role in the embryonic development of *Homo sapiens* [20]. The main pathological functions attributed to Twist1 in human cancers are related to invasion and metastasis by promoting EMT in solid tumors [8]. In BC, Twist1 is described as the master regulator of metastasis [9]; however, several issues involving its role in different types and stages of human cancers remain to be addressed, particularly in BC subtypes. The present study demonstrated the strong association between Twist1 and IL-17 signaling in HER2+ BC, suggesting a role for Twist1 in immune processes, beyond EMT.

Initially, the expression of *TWIST1*, *SLUG*, and *SIP1* was evaluated individually and compared to patient data from the Brazilian National Cancer Institute cohort, which is a strictly selected cohort of invasive ductal carcinoma (IDC) containing all the relevant clinicopathological information (Table 2). Although the TNBC group has been more strongly associated with the EMT expression profile [21], we found significantly increased expression of *TWIST1* in HER2+ BC samples (Figure 1), which is in accordance with Qiao et al. [22].

Interestingly, we observed a lower difference of *TWIST1* levels in HER2+ BC samples from the TCGA database (Figure 2) compared to the Brazilian group, which can be explained by the ethnic genetic variability between these two cohorts. Byun et al. [23] demonstrated that the relevance of the master regulators of Luminal BC, FOXA1 and GATA3, to patient survival is different depending on the ethnicity of the patients. More recently, Romero-Cordoba et al. [24] reported results showing that the molecular features of BC in a set of Hispanic-Mexican women are significantly distinct from Caucasians, which supports the relevance of studying the Hispanic/Latino BC patients that share similar genomic basis with Native American, European, and African ancestries.

Since high-throughput strategies provide a powerful tool for investigating molecular alterations, we sought to evaluate which biological processes are related to Twist1 in HER2+ cells. After confirming the *TWIST1* knockdown (Figure 3), we proceeded to transcriptomic analyses combined with in silico analyses using the MetaCore^TM^ software. *TWIST1* knockdown altered important biological processes related to cellular homeostasis. EMT was the most statistically significant altered biological process (Table 1, Figure S2), which confirmed the relevance of Twist1 to this phenomenon in BC. We observed the upregulation of *ZO3* and *CLDN8*, which encode tight junction protein 3 and claudin-8, essential proteins to epithelial organization (details of the signaling pathway on Figure S3). We also found increased expression of KTR4 in *TWIST1*-knockdown cells, which encodes for the epithelial differentiation marker cytokeratin 4. Consistently, we observed the downregulation of genes that encode for the EMT-inducing factors HMGA2 and SLUG, as well as for the fibronectin gene (*FN1*), a glycoprotein that supports cell migration. These data are in accordance with the well-documented role of Twist1 in the repression of epithelial markers [25,26] and the activation of mesenchymal markers [27,28,29] such as SLUG, another EMT-inducing TF [30].

We found the altered expression of genes related to ECM remodeling and blood coagulation in *TWIST1*-knockdown cells (details of the signaling pathway on Figure S4). Interestingly, most of the identified genes in this group are relevant members of the angiogenesis and blood clotting cascades, including several serine-protease enzymes. Twist1 is commonly associated with angiogenesis in cancer [31,32], although the regulatory targets of Twist1 in this context have yet to be determined. Additionally, we observed the upregulation of *PKCA* (protein kinase Cα), which is described as inducing endothelial nitric oxide synthase (eNOS) and blood flux, and *IP3R1,* the encoded protein of which, inositol 1,4,5 triphosphate receptor acts on vasoconstriction dependent on Ca^2+^. Regarding the regulation of serine-protease enzymes, Twist1 is associated with the upregulation of MMPs and the repression of their inhibitors [33]. Our results showed the upregulation of *SERPINA1*, which encodes the protease inhibitor α1-antitrypsin, in *TWIST1* knockdown cells. Conversely, we observed the upregulation of *PLAT*, which encodes the tissue plasminogen activator (t-PA), responsible for activating plasmin and MMPs during blood clotting and ECM remodeling, and the downregulation of *PAI1* and *PAI2*, which are inhibitors of t-PA activity. Although *HB-EGF* was grouped with ECM remodeling genes by the Metacore^TM^ software, its downregulation is more related to a probable HER2/NF-κB/Twist1 axis. HB-EGF mimics EGF in EGFR signaling, and since HER2+ BC was demonstrated to induce NF-κB [34], which activates *TWIST1* expression [17], HB-EGF might support a positive feedback loop in HER2+ BC.

Considering our microarray dataset, the most interesting results involved the downregulation of relevant members of the IL-17 signaling pathway following *TWIST1* knockdown. The IL-17 family of cytokines is composed of six members (IL-17A, IL-17B, IL-17C, IL-17D, IL-17E, and IL-17F) that mediate innate and proinflammatory responses and are produced by different cell types. These cytokines are secreted as homo or heterodimers and bind to members of the IL-17R family (IL-17RA, IL-17RB, IL-17RC, IL-17RD, and IL-17RE). IL-17 cytokines have a higher affinity for specific receptors and oligomerize to form functional complexes [35,36,37]. The binding of IL-17 ligands to their receptors leads to the recruitment of the adaptor protein Act1, which leads to signaling transduction and the expression of IL-6, IL-8 and antimicrobial peptides [37,38]. The mechanism of Th17 differentiation involves the activation of the transcription factor RORγt, which stabilizes the Th17 phenotype, and is the master transcriptional regulator of Th17 cells.

As shown in Figure S5, we observed the downregulation of the chemotactic factors CCL20 and GM-CSF, and of the Th17 differentiation factors IL23A and IL-6. For a better understanding of IL-17 signaling, we included relevant members of this signaling pathway in the RT-qPCR analysis. We observed the downregulation of the mRNA levels for *IL17RA, IL17A, IL17F, IL23A, CCL20, IL6, IL8*, and *RORγt/RORC2* in *TWIST1* silenced cells compared to control cells (Figure 4C and Figure 5). Strengthening our data, we confirmed the diminished expression of *IL17RA* and *ACT1* at the protein level (Figure 6A) as well as the reduced secretion of IL-6 and IL-8 (Figure 6B) following *TWIST1* knockdown. Thus, we observed that *TWIST1* silencing affected the main receptor, the hallmark cytokines and the master transcriptional regulator of the Th17 phenotype in HER2+ BC cells. These results indicated that Twist1 cooperates with IL-17 signaling in HER+ BC cells. The induction of IL-6 and IL-8 expression by IL-17 activation has already been described in other nonimmune cell types such as fibroblasts and epithelial cells [36], but not in BC so far.

Contradictorily, Pham et al. [39] demonstrated that Twist1 represses the IL6-STAT3 axis, impairing the differentiation of the CD4 T helper cell into a Th17 cell, while [40] showed the role of IL-17 in inducing EMT in lung adenocarcinoma cells. Thus, to the best of our knowledge, our study is the first time that the link between Twist1 and IL-17 signaling in HER2+ BC has been reported. However, functional assays to investigate the relevance of the proposed axis need to be further explored in future studies.

Due to the relevance of RORγt to the Th17 phenotype, we investigated its expression in BC samples from the Brazilian National Cancer Institute cohort and found it to be overexpressed in HER2+ BC (Figure 7A), similar to *TWIST1* expression (Figure 1A). We also performed a correlation analysis of mRNA levels (*p* = 0.0928, Figure 7B), which supported our hypothesis proposing a link between Twist1 and IL-17 signaling in HER2+ BC. Since *RORC2* (RORγt) is a variant, we were not able to evaluate its expression through the Linkedomics/TCGA database; however, we performed analyses for IL17RA, IL17RC, IL17A and IL17F (Figure 8 and Figure 9), which agreed with our findings that showed the downregulation of IL17A and the upregulation of IL17RC in *TWIST1* silenced cells (Figure 4C and Figure 5). Cao et al., reported that Twist1 up-regulates *ROR1* in basal-like BC [41]. It is reasonable that the authors identified this association in TNBC, since ROR1 acts as a receptor of Wnt5a in the noncanonical pathway of WNT signaling in this subtype [42]. Although ROR1 and RORγt belong to the ROR subfamily, RORγt has an entirely different role, acting as a TF of IL-17 members.

Although IL-17 signaling plays a critical role in eliminating extracellular pathogens [36], this signaling may also contribute to the pathogenesis of autoimmune diseases including psoriasis and rheumatoid arthritis [43,44]. The role of this signaling in cancer is controversial. While IL-17A acts as a potent inducer of T-cell-mediated antitumor immune responses, this cytokine was described as an essential activator of angiogenic factors, as well as an inducer of the proinflammatory factors IL-6 and IL-8 in cancer [35,45,46]. In addition, therapies targeting IL-17 have been proposed for non-small cell lung cancer [47], although nothing has been suggested for BC so far.

Although there are indications of a probable association between Twist1 and IL-17 in hematopoietic cancers, this association was poorly evidenced in tumors from epithelial cells. In summary, the present study demonstrated for the first time the role of Twist1 on IL-17 signaling in HER2+ BC, which sheds new light on inflammation and EMT in this subtype specifically. Thus, we suggest that the findings described in this study could improve understanding of the role of Twist1 in BC biology and emphasize Twist1 as a potential target for the development of target-specific therapies.

## 4. Materials and Methods

### 4.1. Patient Samples

Human samples were obtained from BC patients from the Brazilian National Cancer Institute (Rio de Janeiro, Brazil) under the approval of the Institutional Ethics Committee, and all procedures were conducted according to the principles from the Declaration of Helsinki. All patients enrolled signed informed consent forms. Clinical data were obtained from medical records including clinicopathological parameters, information concerning treatment and follow-up (Table 2, all records were reviewed.)

BC patients diagnosed with unilateral primary iinvasive ductal carcinoma (IDC) were included in this study. Considering that therapies may influence gene expression, none of the patients had received chemo or radiotherapy prior to sample collection. The exclusion criteria used were as follows: evidence of a diagnosis of cancer other than BC; male BC; bilateral BC tumors; other histological types than IDC; any hormone/chemotherapy and/or radiotherapy before tissue collection; insufficient information on clinicopathological parameters; loss of follow-up, and no fluorescence in situ hybridization (FISH) data for reported HER2 intermediate staining. The TNM staging system was used following the eighth edition of the AJCC Cancer Staging Manual [48]. Lymph node status was determined by pathological examination. The BC subtype classification was based on immunohistochemistry (IHC) data. The cutoff for ER or PR positivity was 1% detection in the nucleus [49]. HER2 status was considered positive when strong membranous staining was observed or when confirmed by FISH when the IHC result was intermediate [50]. TNBCs were considered based on the lack of staining of the markers described above.

### 4.2. Cell Culture and Lentiviral Transduction

The human BC cell lines HCC-1954 (CRL-2338) and SK-BR-3 (HTB-30) are ER-, PR-, and overexpress HER-2/neu. HCC-1954 cells were cultured as previously described [17]. SK-BR-3 cells were cultured in RMPI-1640 (Sigma-Aldrich, St. Louis, MO, USA) supplemented with 10% fetal bovine serum (FBS), 0.4% glucose, 2 mM glutamine, 100 U/mL penicillin and 100 μg/mL streptomycin. The cells were cultured in a humidified 5% CO_2_ atmosphere at 37 °C.

To knockdown *TWIST1* (NM_000474.3) in HCC-1954 cells, short hairpin RNA (shRNA) was used (SHCLNV-NM_000474, Sigma-Aldrich, St. Louis, MO, USA). A nontarget shRNA (ShCTRL, SHC002V, Sigma-Aldrich, St. Louis, MO, USA) was used as a negative control. Lentiviral transductions were performed according to the manufacturer’s instructions (MISSION Lentiviral Transduction Particles, Sigma-Aldrich). The structure of the vector used was the pLKO.1 plasmid containing the hairpin coding sequence, puromycin resistance gene, and green fluorescent protein (GFP) gene. Transduced cells were selected by incubation with 500 ng/mL of puromycin (Sigma-Aldrich. St. Louis, MO, USA) for two months, and the percentage of GFP-positive cells was routinely evaluated by flow cytometry (FACSCalibur, BD Biosciences, San Jose, CA, USA).

To knockdown *TWIST1* in SK-BR-3 cells, specific short interference RNA (siRNA) (s14523, Ambion, Austin, TX, USA) was used. An oligonucleotide that did not match any human coding cDNA was used as a nontargeting control (siNT, sc-37007, Santa Cruz Biotechnology, Dallas, TX, USA). Transfections were performed using Lipofectamine RNAiMAX Reagent as per the manufacturer’s instructions (Invitrogen, Waltham, MS, USA).

### 4.3. Expression Chip Array Data Analysis

Total RNA from *TWIST1*-silenced (ShTWIST) and nonsilenced cells (ShCTRL) were obtained with the RNeasy Mini kit (Qiagen, Hilden, Germany) following the manufacturer’s instructions. Next, 100 ng of total RNA was used to synthesize and biotinylated cRNA according to the GeneChip whole transcription sense target labeling assay (Thermo Fisher, Waltham, MS, USA). The biotinylated cRNA was hybridized to GeneChip Human Exon 1.0 ST arrays (Thermo Fisher), washed and stained according to the manufacturer’s protocols. The GeneChip arrays were scanned using a GeneChip^®^ Scanner 3000. Data were analyzed using Transcriptome Analysis Console (TAC) software version 4.0 (Thermo Fisher), whereby a ≥2-fold-change was used as the criteria to define upregulation or downregulation of differentially expressed genes compared to expression in ShCTRL. In silico analysis was performed using MetaCore^TM^ software (http://portal.genego.com/; accessed on May 2019) to study gene ontology, biological processes and signaling pathways of differentially expressed genes as a consequence of *TWIST1* knockdown.

### 4.4. Real-Time Reverse Transcription Polymerase Chain Reaction (RT-qPCR)

Total RNA was isolated from cells and patients using TRIzol reagent (Thermo Fisher) according to the manufacturer’s instructions. A quantity of 2 μg of RNA was treated with the DNase Amplification Grade I Kit (Thermo Fisher) and reverse transcribed into cDNA using the Superscript-III kit (Thermo Fisher) following the manufacturer’s protocol. RT-qPCR was performed with SYBR Green Master Mix (Thermo Fisher) in a Rotor-Gene Q (Qiagen) under previously reported conditions [17]. The primers used are described in Supplementary Table S1. Mean values of *ACTB* and *GAPDH* housekeeping genes were used as the reference expression for the mRNA levels. Each sample was examined in triplicate. Fold expression was calculated according to the ΔΔC_t_ method [51]. ΔΔCt was calculated against expression in ShCTRL for experiments using cells, while ΔΔCt was calculated against the median of expression in the Luminal group for experiments using BC samples. For survival analysis, patients were classified into high and low-expression groups with the median fold-change for each gene considered to be the cutoff.

### 4.5. Immunoblotting

Protein extracts from cells were obtained as previously reported [17]. The protein concentrations were determined using the Bradford assay [52]. Protein extracts (25 μg) were separated by 10% sodium dodecyl sulfate-polyacrylamide gel electrophoresis (SDS-PAGE), transferred to nitrocellulose membranes (Bio-Rad, Hercules, CA, USA), incubated with anti-Twist1 (SC-81417, Santa Cruz Biotechnology), anti-IL-17RA (FAB177P, R&D Systems, Minneapolis, MI, USA), and anti-Act1 (SC-100647, Santa Cruz Biotechnology) antibodies at a 1:500 dilution at 4 °C overnight, followed by incubation with the appropriate secondary antibody at a 1:2000 dilution at room temperature for 2 h. Anti-GAPDH (SC-47724, Santa Cruz Biotechnology) at a 1:2000 dilution was used as loading control. Antibody binding was detected using enhanced chemiluminescence with Pierce Plus Western Blotting Substrate (Thermo Scientific). Images were obtained using iBright FL1500 Imaging System instrument (Thermo Scientific). Densitometric analysis was conducted using ImageJ 1.53k (NIH). Values obtained for GAPDH staining was used to normalize the densitometry values to the probed targets.

### 4.6. Flow Cytometry

Adherent HCC-1954 cells (ShCTRL and ShTWIST) were processed into single-cell suspensions by trypsin digestion (0.25%, Sigma-Aldrich). Cell-surface staining was performed by incubating the cells with the following antibodies: AlexaFluor 488-conjugated anti-IL-17RC (FAB22691G, R&D Systems) and AlexaFluor 488-conjugated isotype control (IC0041G, R&D Systems), according to the manufacturer’s instructions. Intracellular staining was performed by a fixation step (PBS/Formaldehyde 0.05%) followed by permeabilization (PBS/Tween-20 0.05%) prior to intracellular staining with primary anti-Act1 (WW-18, SC-100647, Santa Cruz Biotechnology) or isotype control (SC-3878, Santa Cruz Biotechnology) antibodies and secondary antibody IgG2a-FITC (sc-358947, Santa Cruz Biotechnology), following the manufacturer’s instructions. Twenty thousand events were collected from each sample. The results were expressed as a percentage of positive cells that was calculated as experiment minus isotype control. All experiments were performed at least three times. Data were acquired using a FACSCalibur flow cytometer and analyzed using CellQuest software version 5.2 (BD Biosciences, San Jose, CA, USA).

### 4.7. Light Phase Contrast and Fluorescence Microscopy

Images of ShCTRL and ShTWIST cells were acquired (200× magnification) using an Axio Observer.Z1 microscope equipped with an AxioCam HRc and the AxioVision digital image processing software version 4.8 (Carl Zeiss Inc., Thornwood, NY, USA).

### 4.8. Enzyme-Linked Immunosorbent Assay (ELISA)

Interleukin (IL)-6 and IL-8 levels in supernatant cultures were evaluated using a commercial antibody-specific ELISA kit (Peprotech, East Windsor, NJ, USA), according to the manufacturer’s instructions. For both ShCTRL and ShTWIST cells, four different conditions were analyzed: (1) nontreated cells, (2) incubation with human recombinant IL-17A (50 ng/mL, Peprotech), (3) incubation with IL-17F (50 ng/mL, Peprotech), and (4) incubation with IL-17A and IL-17F simultaneously (25 ng/mL each). The experiments were performed in triplicate and analyzed with an ELISA microplate reader at 450 nm (Asys Expert Plus, Biochrom, Cambridge, UK). The results are represented as the ratio of the experiments over the control.

### 4.9. TCGA Analysis

We used the the LinkedOmics portal (http://www.linkedomics.org/login.php; accessed on May 2021) to access and conduct the gene expression analysis at the mRNA level from The Cancer Genome Atlas (TCGA) patients’ database. LinkedOmics has three modules: LinkInterpreter, LinkFinder and LinkCompare. The Linkfinder module was used to obtain data of differentially expressed genes and to identify the coexpressed genes related to TWIST1 in Breast invasive carcinoma (TCGA-BRCA) cohort (*n* = 1093). RNAseq (HiSeq, Gene level) data were used: RNAseq data normalized counts (Illumina HiSeq platform, Gene-level, Reads Per Kilobase of transcript, per Million mapped reads (RPKM)) gene Expression (RNA-Seq by Expectation-Maximization (RSEM), Log2(Val+1)). Results of coexpression were assessed using Spearman correlation analysis for statistical analysis.

### 4.10. Statistical Analysis

Categorical variables were expressed as percentages, while numerical variables were expressed as the mean ± standard deviation (SD) or median (minimum-maximum). We used the Kolmogorov-Smirnov test to examine whether variables were normally distributed. The Kruskal-Wallis test was used to compare numerical variables among three or four groups, and the Mann-Whitney test was performed for comparison between two groups. Spearman correlation tests were used to correlate continuous numeric variables among groups. Kaplan-Meier’s analysis was used to assess death risk according to gene expression. The Wilcoxon test was used to compare survival distribution. Gene expression results were presented by at least three independent experiments and analyzed by a two-tailed Student’s *t*-test or ANOVA. Statistical analysis was performed using SPSS v.20.0 (IBM SPSS Statistics, Armonk, NY, USA) and GraphPad Prism v.5 (GraphPad Inc., San Diego, CA, USA). *p*-values < 0.05 (two-sided) were considered statistically significant (* = *p* < 0.05; ** = *p* < 0.01; *** = *p* < 0.001), and post hoc analyses (Tukey’s honest significance or Dunn’s multiple comparison tests) were applied when necessary.

## Figures and Tables

**Table 1:** Biological processes related to differentially expressed genes following TWIST knockdown.

Functional Enrichment Analysis *	Gene #	
	Upregulated	Downregulated
EMT/Cell adhesion	*ZO3, CLDN8, KTR4*	*TUBA3C, FN1, SLUG, HMGA2*
ECM remodeling/Blood coagulation	*IP3R1, PLAT, SERPINA1, PKCA, ITGB3, GNAI2*	*HBEGF, PAI1, PAI2*
Immune response/Th17-derived cytokines		*IL23A, IL6, IL8, CCL20, CSF2*

* Enrichment analysis was performed using MetaCore^TM^ software (http://portal.genego.com/; accessed on May 2019); # Symbols of representative genes identified to be upregulated or downregulated in pathway maps; EMT = epithelial-mesenchymal transition; ECM = extracellular matrix.

**Table 2:** Clinicopathological characteristics of patients.

Clinical Characteristic	*n* = 46	% = 100
**Age, yr**		
Early onset (20–50) Late onset (51–80)	17 29	37.0 63.0
**Female, *n***	46	100
IHC-based molecular classification *		
Luminal A Luminal B HER2-enriched Triple Negative	16 11 8 11	34.8 23.9 17.4 23.9
**Tumor histological type**		
Invasive ductal carcinoma	46	100
**Histological grade**		
Grade 1 Grade 2 Grade 3	3 8 35	6.5 17.4 76.1
**TNM staging**		
I/II III/IV	31 15	67.4 32.6
**Tumor largest diameter, cm**		
<2 2–5 >5	9 35 2	19.6 76.1 4.3
**Lymph node status, *n***		
Stage 1 (negative) Stage 2 (1–3 LN) Stage 3 (>3 LN)	17 20 9	37.0 43.5 19.6
**Distant metastasis, *n***		
Presence Absence	10 36	21.7 78.3

* According to Voduc, et al. [2]; IHC = Immunohistochemistry; LN = Lymph node.

## Data Availability

The data is provided as supplementary material.

## Supplementary Materials

The following are available online at https://www.mdpi.com/article/10.3390/ijms222212144/s1.
