# Supplementary material for: Twist1 Influences the Expression of Leading Members of the IL-17 Signaling Pathway in HER2-Positive Breast Cancer Cells
Source: Int J Mol Sci. 2021 Nov 10;22(22):12144. doi: 10.3390/ijms222212144 (PMC8620489; doi:10.3390/ijms222212144)
Supplement: Supplementary file 1 [file ijms-22-12144-s001.zip › Supplementary_Pires et al.pdf]

SUPPLEMENTARY MATERIAL

To study the molecular changes observed as a consequence of *TWIST1* silencing compared to control cells, we conducted *in silico* analyses using MetaCore^{TM} software (http://portal.genego.com/). This software grouped differentially expressed genes according to gene ontology (Supplementary Figure S1), biological processes (Supplementary Figure S2), and signaling maps (Supplementary Figures S3-S5).

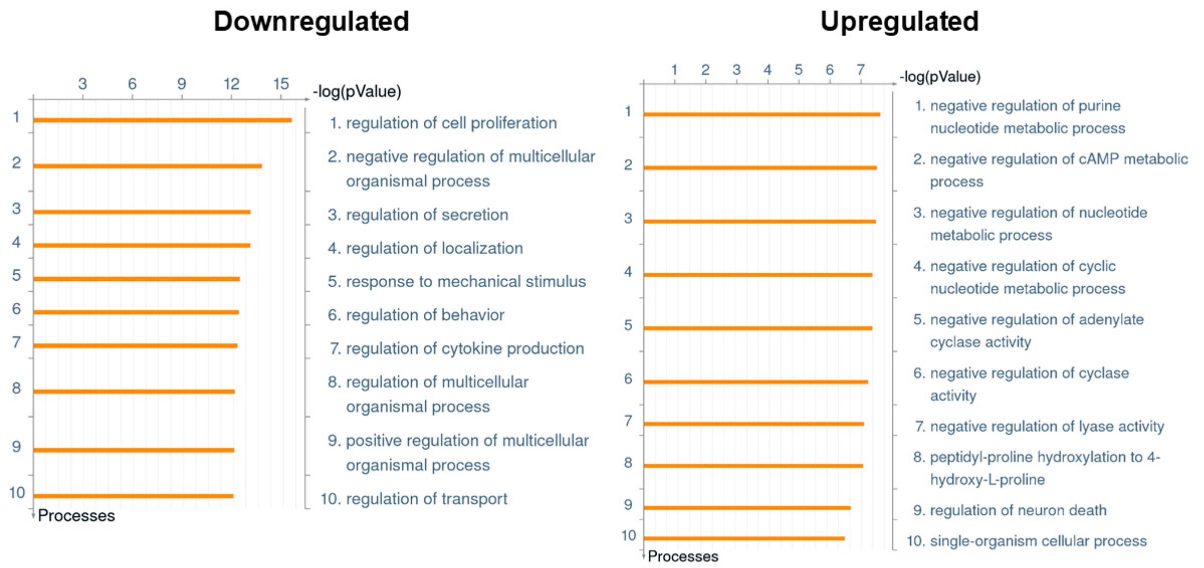

Supplementary Figure S1. Gene ontology of the downregulated and upregulated transcripts as a result of *TWIST1* silencing. The processes are ordered according to the statistical significance of the genes presented in each condition.

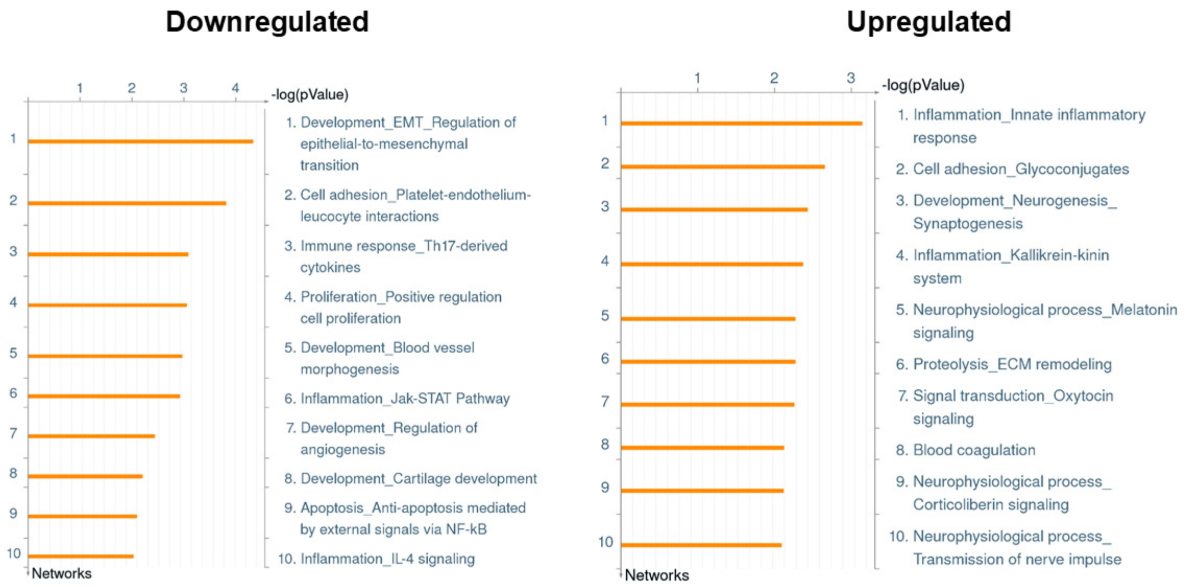

Supplementary Figure S2. Biological processes altered in downregulated and upregulated transcripts as a result of *TWIST1* silencing. The processes are ordered according to the statistical significance of the genes presented in each condition.

A

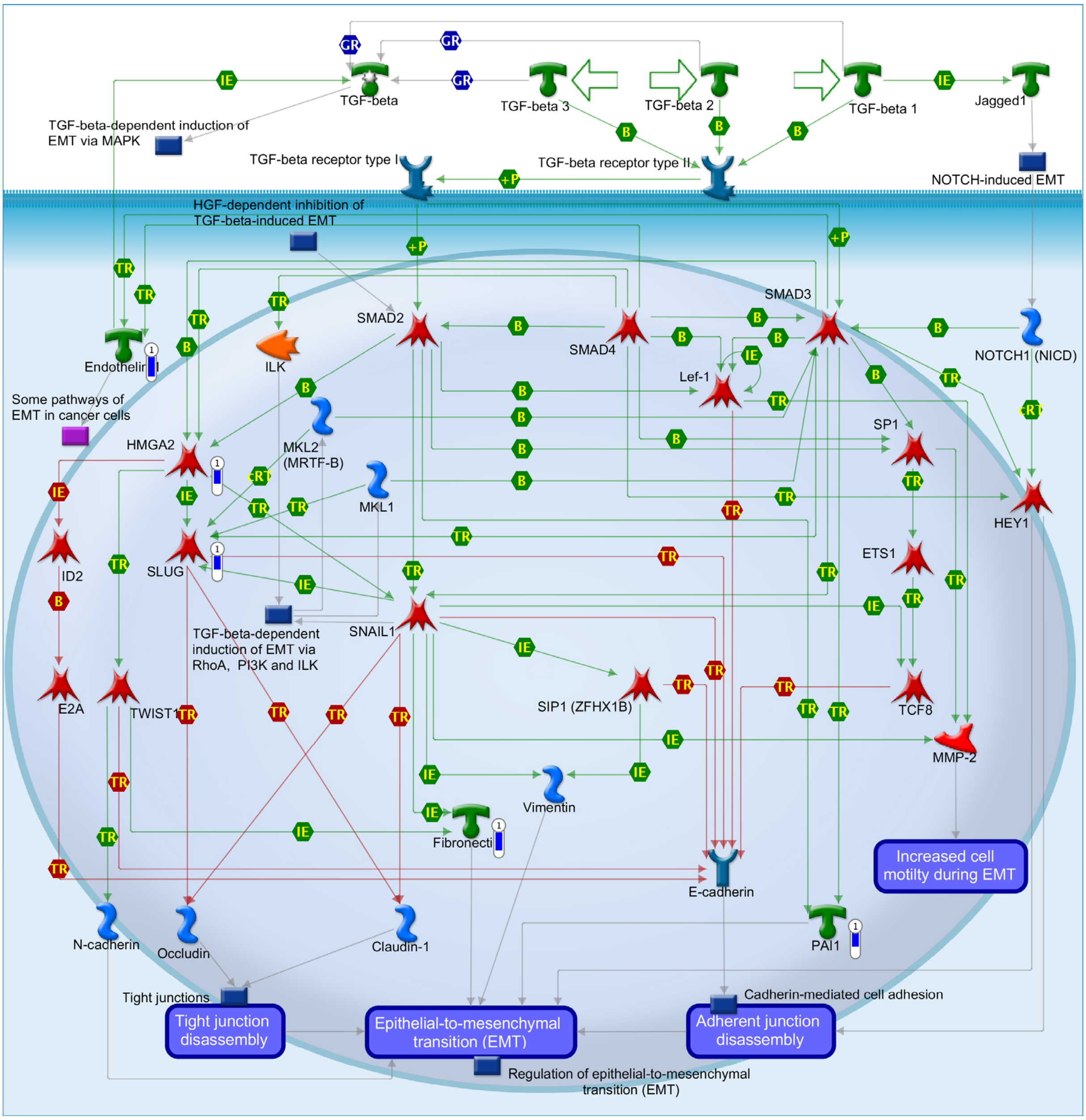

B

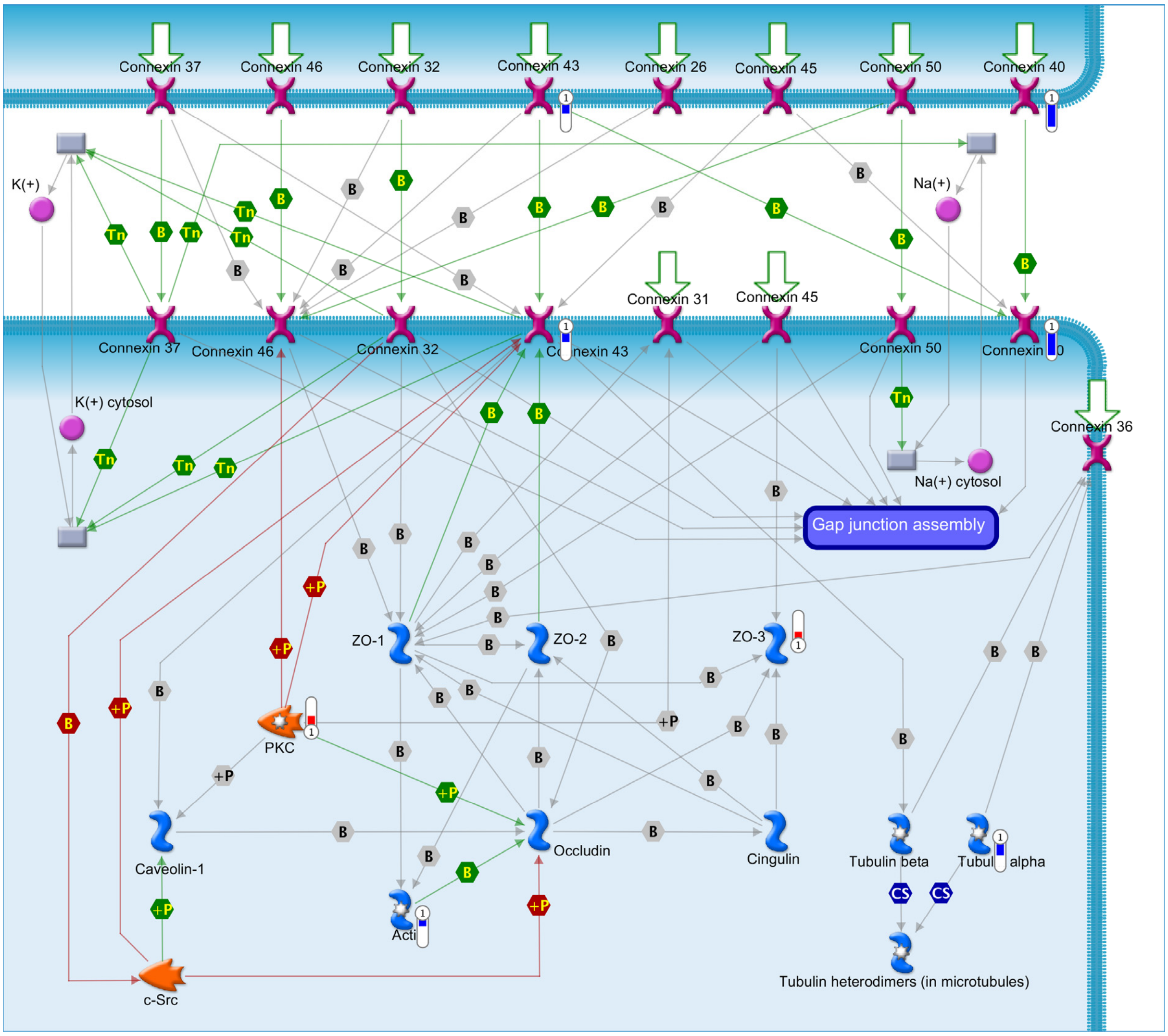

Supplementary Figure S3. "Regulation of Epithelial-Mesenchymal Transition" (A) and "Cell adhesion" (B) were the top scored maps altered as the result of *TWIST1*-silencing based on the enrichment distribution of genes presented in the analyses. The relative gene expression data are visualized on the map by thermometer-like figures in blue (for downregulation) or red (for upregulation).

A

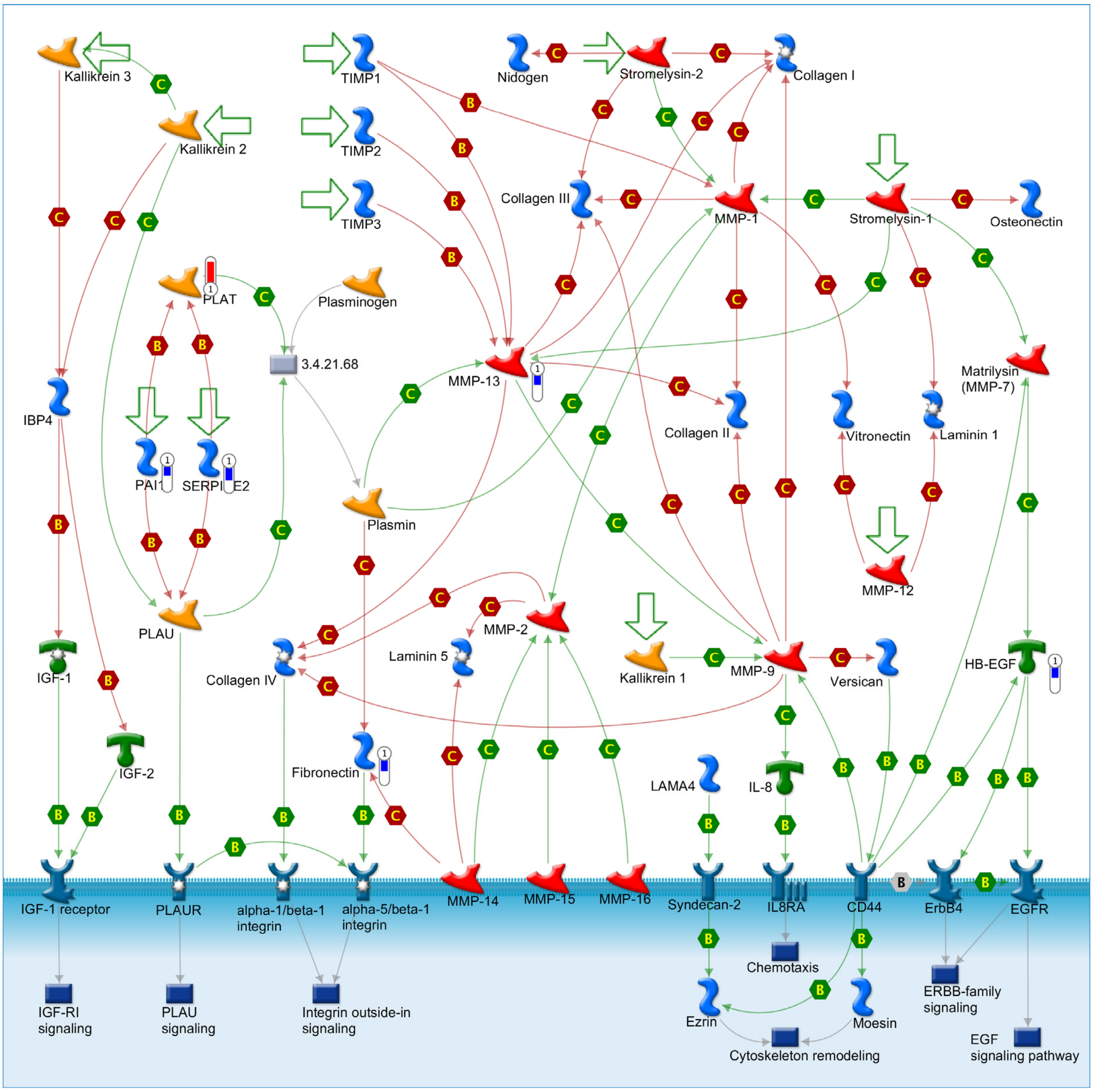
Kallikrein 3
Kallikrein 2
TIMP1
TIMP2
TIMP3
Nidogen
Stromelysin-2
Collagen I
Collagen III
MMP-1
Stromelysin-1
Osteonectin
PLAT
Plasminogen
3.4.21.68
IBP4
MMP-13
Matrilysin (MMP-7)
Collagen II
Vitronectin
Laminin 1
PAI1
SERPINE2
Plasmin
MMP-12
PLAU
MMP-2
Laminin 5
Collagen IV
Kallikrein 1
MMP-9
Versican
HB-EGF
IGF-1
IGF-2
Fibronectin
LAMA4
IL-8
IGF-1 receptor
PLAUR
alpha-1/beta-1 integrin
alpha-5/beta-1 integrin
MMP-14
MMP-15
MMP-16
Syndecan-2
IL8RA
CD44
ErbB4
EGFR
IGF-RI signaling
PLAU signaling
Integrin outside-in signaling
Chemotaxis
Ezrin
Moesin
Cytoskeleton remodeling
ERBB-family signaling
EGF signaling pathway

B

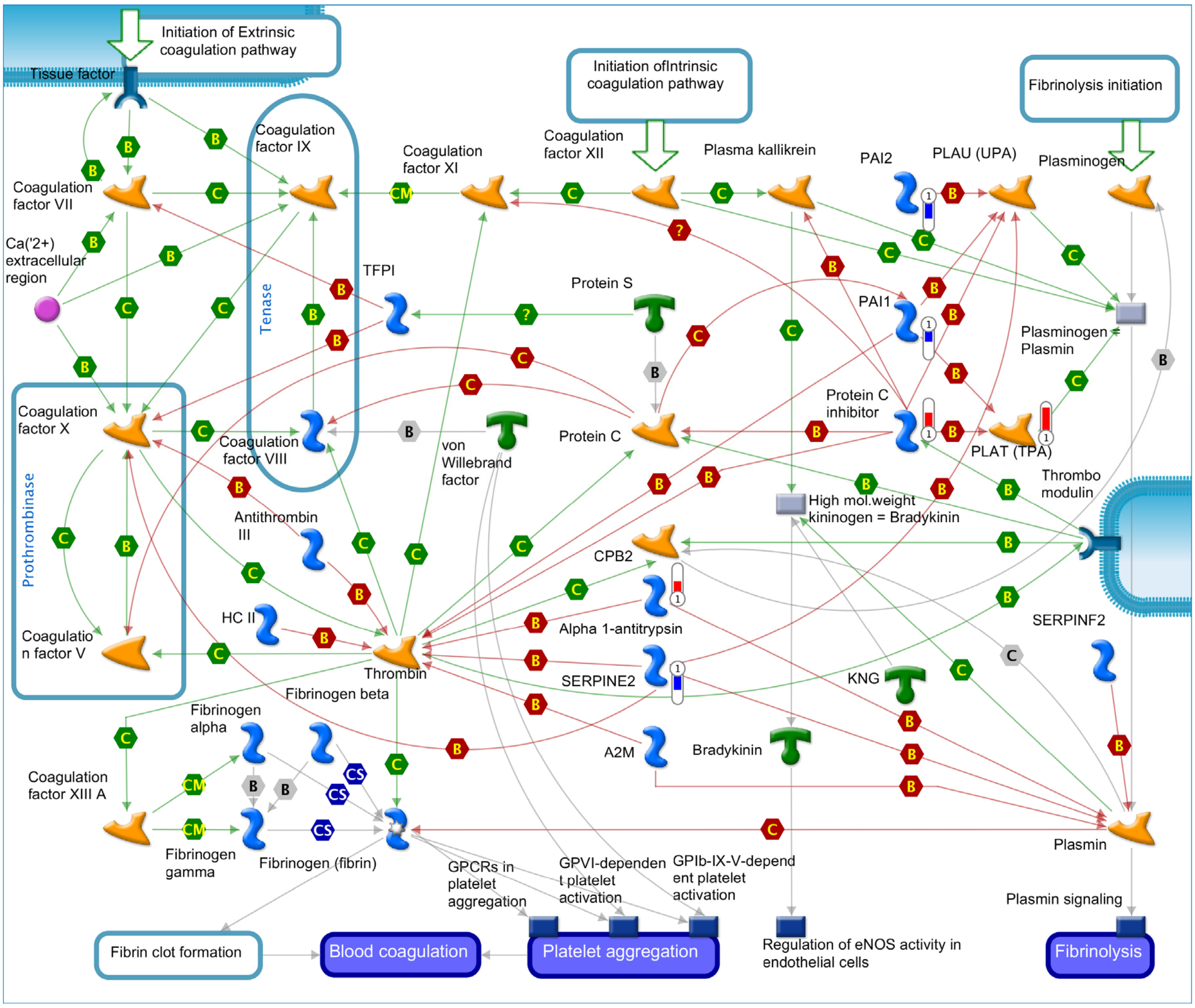

Supplementary Figure S4. "ECM remodeling" (A) and "Blood coagulation" (B) were the second scored maps altered as the result of *TWIST1* silencing based on the enrichment distribution of genes presented in the analyses. The relative gene expression data are visualized on the map by thermometer-like figures in blue (for downregulation) or red (for upregulation).

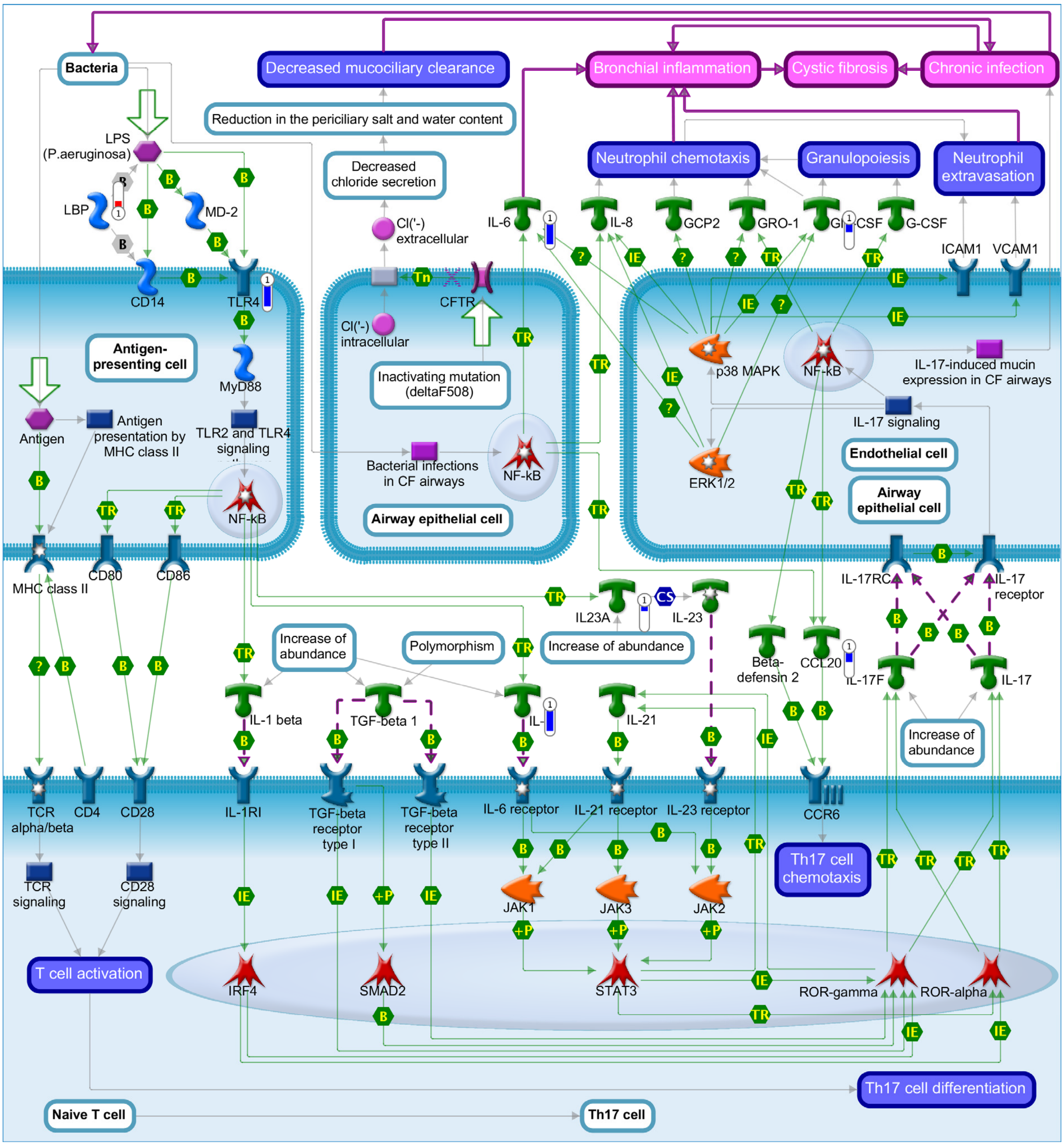

Supplementary Figure S5. "Th17-mediated Immune Response" was the third scored map altered as the result of *TWIST1* silencing based on the enrichment distribution of genes presented in the analyses. The relative gene expression data are visualized on the map by thermometer-like figures in blue (for downregulation) or red (for upregulation).

Supplementary Table S1. Primer sequences of the investigated genes.

Primer	Sequence
ACTB forward	5’-TTC CTT CCT GGG CAT GGA GTC-3’
ACTB reverse	5’-AGA CAG CAC TGT GTT GGC GTA-3’
GAPDH forward	5’-ACC CAC TCC TCC ACC TTT GA-3’
GAPDH reverse	5’-CTG TTG CTG TAG CCA AAT TCG T-3’
NF-κB(RELA) forward	5’-CTA CGA CCT GAA TGC TGT GC-3’
NF-κB(RELA) reverse	5’-CTG CCA GAG TTT CGG TTC AC-3’
TWIST1 forward	5’-GGC ACC ATC CTC ACA CCT CT -3’
TWIST1 reverse	5’-TGG CTG ATT GGC ACG ACC T-3’
SLUG forward	5’-AAG CAT TTC AAC GCC TCC AAA-3’
SLUG reverse	5’-GGA TCT CTG GTT GTG GTA TGA CA-3’
SIP1(ZEB2) forward	5’-AAA CAA GCC AAT CCC AGG AG-3’
SIP1(ZEB2) reverse	5’-GCA CAC TAG CTG GAC TCG TCT C-3’
HBEGF forward	5’- GCT GTG GTG CTG TCA TCT GT -3’
HBEGF reverse	5’- CAT GCC CAA CTT CAC TTT CTC -3’
IL6 forward	5’- GGT ACA TCC TCG ACG GCA TC -3’
IL6 reverse	5’- GCC TCT TTG CTG CTT TCA CAC -3’
THBS1 forward	5’- AGA CTC CGC ATC GCA AAG G -3’
THBS1 reverse	5’- GCA GCC TTT GTT CCT GAG GA -3’
TUBA3C forward	5’- CGC ACC ATC CAG TTT GTA GA -3’
TUBA3C reverse	5’- GTG TTG CTC AGC ATG CAC AC -3’
PAI1 forward	5’- CAA GAG CCT CTC CAC GTC G -3’
PAI1 reverse	5’- CGG ACC ACA AAG AGG AAG GG -3’
PAI2 forward	5’- CTC AGA ACC CCA GGC AGT AG -3’
PAI2 reverse	5’- ACA GCA TTC ACC AGG ACC AT -3’
ZO3 forward	5’- GAC AGG ACA GCA TGC GAA CC -3’
ZO3 reverse	5’- CCC CAG TCA TAG CCG TCT TC -3’
PLAT forward	5’- TCA CCG ACA ACA TGC TGT G -3’
PLAT reverse	5’- AGC TGA TGA TGC CCA CCA AA -3’
SERPINA1 forward	5’- AAG GTC TTC AGC AAT GGG GC -3’
SERPINA1 reverse	5’- TTC TCG TCG ATG GTC AGC AC -3’
KRT4 forward	5’- GAC AGC GTG GAG GAC TTC AA -3’
KRT4 reverse	5’- GCC TCC AAC TCC ACC TTG TT -3’
CLDN8 forward	5’- CGT GAG GCA GGC TAA CAT CA -3’
CLDN8 reverse	5’- AGC AGC ACA CAT CAG TCC TC -3’
IP3R1 forward	5’- CTG GCC AGC TGT CGG AAT TA -3’
IP3R1 reverse	5’- GCT GGT TGT TGT GGG TTG AC -3’
PKCA forward	5’- AGA GAG CAT GCC TTC TTC CG -3’
PKCA reverse	5’- GGC TGT CCT CGT GTG AAG AA -3’
IL8 forward	5’- GTG CAG AGG GTT GTG GAG AAG -3’
IL8 reverse	5’- CCC TAC AAC AGA CCC ACA CAA -3’
IL23A forward	5’- AGA AGC TCT GCA CAC TGG C -3’
IL23A reverse	5’- TCA CAG CCA TCT CCA CAC TG -3’
ACT1 forward	5’- CAT CCC TGT GCT CTT CCC AA -3’
ACT1 reverse	5’- TTC TTG GGC CAG CTG TAG AC -3’

CCL20 forward	5’- ACA CAG ACC GTA TTC TTC ATC CT -3’
CCL20 reverse	5’- TTG CGC ACA CAG ACA ACT TT -3’
IL17RC forward	5’- CGA TGA CTT GGG AGC GCT AT -3’
IL17RC reverse	5’- AGC GGC AAA GAG TAG GCA G -3’
IL17RA forward	5’- GTA CTG GTT CAT CAC GGG CA -3’
IL17RA reverse	5’- GCA GGC CAT CGG TGT ATT TG -3’
IL17A forward	5’- ACC AAT CCC AAA AGG TCC TC -3’
IL17A reverse	5’- GGG GAC AGA GTT CAT GTG GT -3’
IL17F forward	5’- CCT CCC CCT GGA ATT ACA CT -3’
IL17F reverse	5’- TTC CTT GAG CAT TGA TGC AG -3’
RORC2 forward	5’- TGG ACC ACC CCC TGC TGA GAA GG -3’
RORC2 reverse	5’- CTT CAA TTT GTG TTC TCA TGA CT -3’
